# Use of Instagram as an Educational Strategy for Learning Animal Reproduction

**DOI:** 10.3390/vetsci12080698

**Published:** 2025-07-25

**Authors:** Carlos C. Pérez-Marín

**Affiliations:** Department of Animal Medicine and Surgery, Campus of Rabanales, University of Cordoba, 14071 Cordoba, Spain; pv2pemac@uco.es; Tel.: +34-957-218-716

**Keywords:** social media, animal reproduction, veterinary education, teaching, learning, Instagram

## Abstract

The present study explores the use of Instagram as an innovative strategy in the teaching–learning process in the context of animal reproduction topics. In the current era, with digital technology and social media transforming how information is accessed and consumed, it is essential for teachers to adapt and harness the potential of these tools for educational purposes. This article delves into the need for teachers to stay updated with current trends and the importance of promoting digital competences among teachers. This research aims to provide insights into the benefits of integrating social media into the educational landscape. Students of Veterinary Science degrees, Master’s degrees in Equine Sport Medicine as well as vocational education and training (VET) were involved in this study. An Instagram account named “UCOREPRO” was created for educational use, and it was openly available to all users. Instagram usage metrics were consistently tracked. A voluntary survey comprising 35 questions was conducted to collect feedback regarding the educational use of smartphone technology, social media habits and the UCOREPRO Instagram account. The integration of Instagram as an educational tool was positively received by veterinary students. Survey data revealed that 92.3% of respondents found the content engaging, with 79.5% reporting improved understanding of the subject and 71.8% acquiring new knowledge. Students suggested improvements such as more frequent posting and inclusion of academic incentives. Concerns about privacy and digital distraction were present but did not outweigh the perceived benefits. The use of short videos and microlearning strategies proved particularly effective in capturing students’ attention. Overall, Instagram was found to be a promising platform to enhance motivation, engagement, and informal learning in veterinary education, provided that thoughtful integration and clear educational objectives are maintained. In general, students expressed positive opinions about the initiative, and suggested some ways in which it could be improved as an educational tool.

## Simple Summary

An Instagram account named “UCOREPRO” was created to deliver educational content through microlearning activities, such as short videos and images. The main objective of this study was to analyze its potential as a pedagogical strategy to enhance student learning on animal reproduction. For this purpose, students from Veterinary degrees, Equine Sports Medicine master’s and livestock vocational training courses were involved as followers on the mentioned Instagram account, and a voluntary survey was conducted at the end of the academic year to assess student’s perceptions about this activity. Findings indicate that students highly value the integration of Instagram into the academic experience, citing increased engagement and accessibility. However, concerns were also reported regarding privacy and potential distractions from learning. Participants expressed a clear preference for visual and concise content formats. Overall, the results suggest that Instagram can be a valuable educational resource when thoughtfully integrated, offering a modern and appealing approach to teaching complex scientific topics.

## 1. Introduction

In recent years, the knowledge and information landscape has evolved rapidly, necessitating adaptation from both students and educators. The rise of social networks, commonly referred to as “social media”, has modified how individuals, especially younger generations, access and consume information. This transformation extends to educational settings, where these networks can serve various pedagogical functions, such as content dissemination, teaching–learning interaction and enhancing student motivation [1]. While various social media platforms are available for educational purposes, such as Instagram, TikTok, Twitter, Pinterest, Facebook and YouTube, their effectiveness might vary significantly. In the context of higher education, a recent review examines the use and impact of social media in academic settings [2]. While the COVID-19 pandemic accelerated the adoption of certain digital tools for online education, it also drew attention to others—such as social media—that had not previously been widely used for educational purposes. Currently, blended learning, which combines online and face-to face-instruction, is a growing pedagogical approach that leverages the advantages of digital resources [3].

To enhance student learning and motivation, it is imperative to leverage the potential of new digital devices, such as computers, laptops, tablets and smartphones. Research suggests that students can become dissatisfied and disengaged when exposed to outdated teaching methods, even when relatively traditional technologies, such as PowerPoint, are used [4]. Technology-enhanced learning systems (commonly known as web-based learning or internet-based learning) and, more recently, mobile learning (M-learning) have gained widespread adoption, offering the advantage of access anytime and anywhere. The ubiquity of smartphones and mobile devices, with approximately 6.8 billion active smartphones and 16.8 billion mobile devices globally in 2023 [5], is indicative of their potential for education. The current portable devices serve not just as a communication tool, but as instruments for social and professional life, and a powerful tool for pursuing academic studies [6].

The transformative role of smartphones, tablets and laptops in medical education has been described elsewhere [7]. However, students often use these devices for non-academic purposes during class. Furthermore, a substantial percentage of students exhibit problematic internet usage, suggesting that the classroom experience needs to be enhanced in order to achieve an appropriate teaching–learning process. A study reports that 68.2% of students were classified as problematic internet users as defined by internet addiction tests, and the use of smartphones in classes was associated with a superficial or low level of learning [8]. The authors of a study related to the use of other portable devices report a similar finding, noting that this trend is associated with the economic status of veterinary students in a range of countries [9]. However, it is important to acknowledge that the implementation of digital learning tools—which depend on technological infrastructure, network connectivity, and access to high-quality digital devices—may exacerbate the digital divide among underserved, deprived, marginalized, and oppressed students [10]. Digital inequities associated with poverty can be observed even in developed countries such as the United Kingdom [11], although they tend to be more pronounced in less developed regions [12]. The global inaction in addressing these disparities risks further widening the gap between students from different countries or socioeconomic backgrounds. This educational paradigm shift, driven by the adoption of innovative technology-enhanced learning tools, has the potential either to bridge or to deepen existing inequalities. Therefore, society as a whole—including stakeholders such as governments, educational institutions, students, educators and citizens—must advocate for digital equity, which is now essential to ensure fair and inclusive access to the emerging landscape of digital education.

Creating digital content is considered a valuable learning task for students. Assigning innovative tasks to students transforms them from passive consumers into active producers of knowledge [13]. A study conducted among students of physical sciences reported that the creation of short videos about the course content can be used as a motivational tool [14]. Digital tools have become essential at all educational levels, and the proliferation of portable devices is crucial for the success of digital education. Within this context, social media plays a significant role in students’ lives as sources of entertainment, but they also offer substantial opportunities for developing educational competencies. The adoption of technologies and online learning resources in veterinary education is rising at an increasing pace [6].

Instagram, created in 2010, is the third most popular social network, trailing only Facebook and YouTube [15]. Over 50% of Instagram users report engaging with humorous content on the platform, while 46% view creative content [16]. The landscape of social media is continuously evolving, and teachers must stay up-to-date with the latest digital trends. Instagram has been integrated into various educational programs, such as language education [17], sport biomechanics courses [18], teaching studies [19] and audio-visual sciences [20,21], among others. Instagram serves as a democratic and inclusive tool that connects people from different countries and social backgrounds, providing equal access to information. Users turn to Instagram not only to satisfy intellectual needs but also to fulfill emotional needs [22].

The European framework DigCompEdu (the European framework for the digital competences of educators) sets out to promote digital competences among educators, focusing on areas such as professional engagement, digital resources, teaching and learning assessment, and fostering learners’ digital competences [23]. Emphasis is placed on the importance of teachers being proficient in the use of digital technologies, identifying appropriate technologies and training students in digital competences [24]. It is recommended that both existing tools, such as social media, as well as future technological developments (which we may not be aware of at this time), are directed towards educational purposes in schools and universities. This involves creating engaging educational content, mainly aimed at and received by millennials, capable of competing with non-educational content. It is equally important to equip teachers with digital competences to create high-quality educational materials. Educators can promote this digital global network to acquire and share knowledge, offer emotional support and build a community [22].

According to the literature reviewed, the use of social media with an educational goal is positively associated with students’ confidence, engagement and creativity; and conversely, cyberbullying, understood as a threatening interaction using the medium of electronic communication, is likely to reduce students’ participation in social media for educational purposes. This research seeks to explore the habits of students in relation to social media, and specifically Instagram, in order to analyze Instagram’s potential as a tool for enhancing the teaching–learning process in the context of animal reproduction topics; it is hoped that this may provide valuable insights into the educational applications of Instagram and its role in modern education.

## 2. Materials and Methods

### 2.1. Study Background

**Figure 1:**
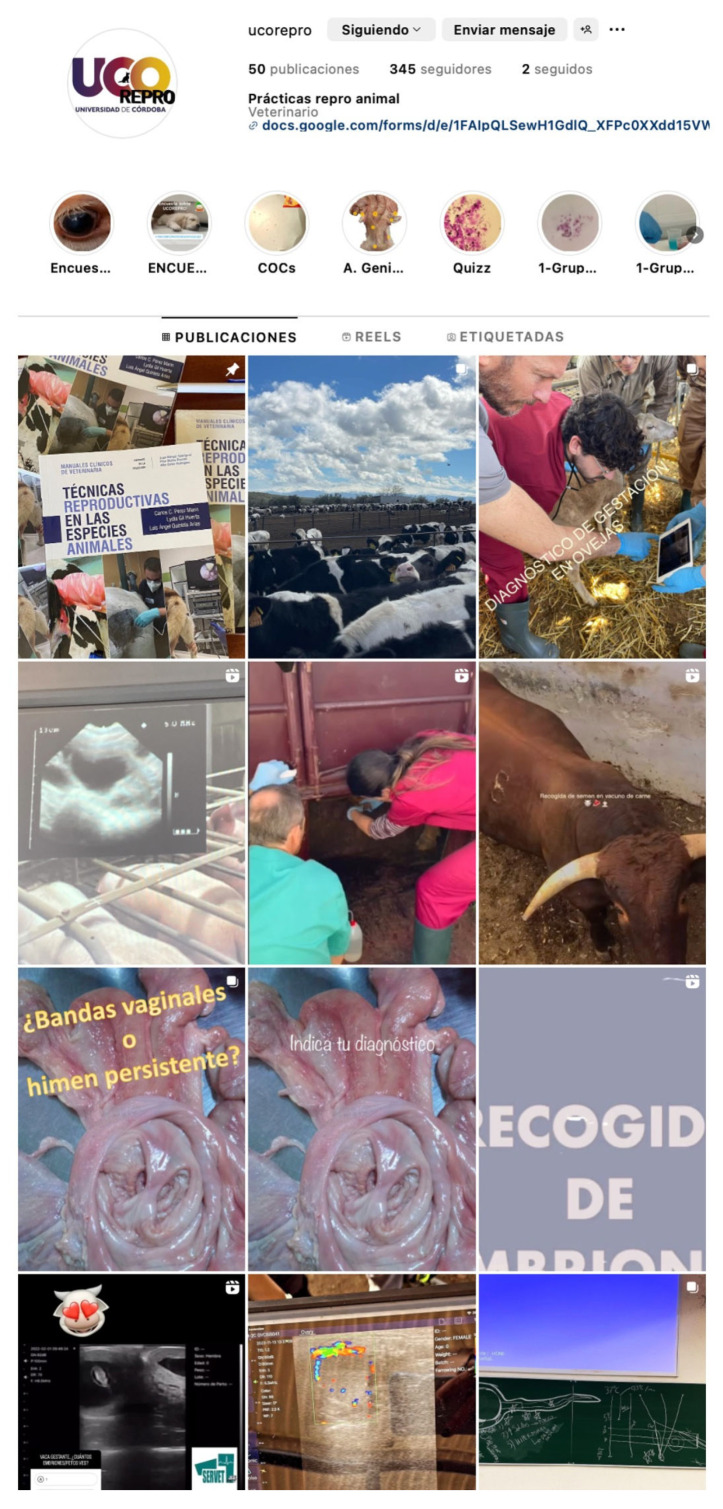
A screenshot illustrating the look of the content on the UCOREPRO Instagram account (https://www.instagram.com/ucorepro/, accessed on 28 May 2025).

The present study was conducted over the course of three academic years. A dedicated Instagram account, “UCOREPRO”, was set up as a training tool for veterinary students (Figure 1). It was created by an academic team from the University of Cordoba (Cordoba, Spain), with a teacher in charge of account creation and management. UCOREPRO emerged as a response to the challenges posed by the COVID-19 pandemic when traditional in-person interactions between teachers and students were limited. This initiative was conceived as a means of providing supplementary academic content and interactions with students during practical sessions. The account was extensively promoted by the teacher among animal reproduction students during the 2020–2021, 2021–2022, and 2022–2023 academic years. It was directed towards fourth-year students enrolled in the animal reproduction module within the Veterinary Science degree at the University of Cordoba, but participation was not mandatory. Access to the account was not restricted to students but was open to all users of the social media platform. Furthermore, students from two other courses, the Master’s in Equine Sport Medicine and vocational education and training (VET), studying husbandry at level 4, were also encouraged to access the account. This Instagram account is being currently used for educational purposes (https://www.instagram.com/ucorepro/, accessed on 28 May 2025).

This study was approved and supported by the Commission for Teaching Training and Educational Innovation at the University of Cordoba (n. 2022-1-3004).

### 2.2. Instagram Use and Survey Preparation

The Instagram account was overseen by a teacher who made decisions about which content was suitable for sharing. To safeguard the privacy of followers, it was determined that the UCOREPRO account would not follow any other Instagram accounts, whether private or otherwise. Content intended for uploading to the Instagram account was created by both the teacher and students, primarily during training and clinical sessions. Approximately 90% of the content was generated by students, and the teacher reviewed all materials before posting to prevent the publication of errors or inconsistent information; explicit consent was obtained to publish educational resources created by the students and/or to use their images. Additionally, quizzes, exercises and other activities were shared with the users. The Instagram metrics were consistently monitored and recorded for each post.

At the end of the 2022–2023 academic year, all UCOREPRO users were invited to participate in a survey canvassing their opinions of the account. All students were also contacted through a virtual learning environment (Moodle), encouraging them to complete the survey. Given the volume of questionnaires that students are required to complete over the years, it was decided to make the survey voluntary. This incurred the risk that insufficient completed surveys would be returned. Although voluntary surveys could be associated with potential bias, it was deemed preferable to collect opinions only from those students who actively participated in these activities and regularly used the UCOREPRO Instagram account. The sample group was considered representative of the studied population.

The survey, comprising a total of 35 questions designed to analyze participants’ social media habits, was administered through Google Forms (see Supplementary Materials). It was also posted on the Instagram account for easy access. In summary, this survey aimed to collect opinions about the use of Instagram as a tool for academic purposes, suggestions for improvement, and an assessment of its advantages and disadvantages. Participants completed these voluntary and anonymous surveys online. The questions were grouped into three sections: “smartphone”, to assess smartphone habits and the use of smartphones for learning purposes; “social media”, to gain insights related to social networks and their use in the learning process; and “Instagram”, which focused on the respondents’ use of the UCOREPRO Instagram account.

Closed questions (multiple choice or yes/no) were used to obtain quantitative data, which are easier to submit to statistical analysis. These were used to gauge student participation and obtain a preliminary snapshot of students’ social media habits and their opinions regarding the potential of Instagram as a learning tool. In addition, some open questions were included to ascertain students’ preferences and perceptions while using Instagram for learning.

### 2.3. Data Analysis

While most questions offered predefined response options, others allowed participants to provide personal opinions. Instagram metrics, such as “story and post views” “likes” “saves” and “accounts reached” were systematically tabulated and analyzed to track changes over time. Data collected from the surveys were processed using descriptive analytical methods provided by Microsoft Excel 16.70 (Microsoft, Redmond, WA 98052, USA).

In order to calculate the internal consistency reliability for measures with dichotomous choices, the Kuder Richardson Formula 20 (KR20) was used:

$$\rho_{\mathrm{KR}20}=\left(\frac{k}{k-1}\right)\left(1-\frac{\sum pq}{\sigma^{2}}\right)$$

where

*k* = number of questions.

*p* = number of participants in the sample who answered “yes”.

*q* = number of people in the sample who answered “no”.

*σ^2^* = variance of the total scores of all the people taking the test.

## 3. Results

### 3.1. Instagram Metrics

By the end of the third academic year (2022–2023), the UCOREPRO account had a total of 252 followers, with a gender distribution of 70.2% women and 29.7% men. This ratio was expected, since veterinary education is known to attract a preponderance of female students, the balance being broadly in line with the aforementioned percentages. It is important to note that the number of followers is subject to fluctuations, and the historical profile of followers cannot be obtained retrospectively. The account displayed consistent activity patterns from 9:00 a.m. to 9:00 p.m., with minimal variation across this time frame. Furthermore, Instagram activity remained constant throughout the week, without any noticeable variations at weekends.

**Figure 2:**
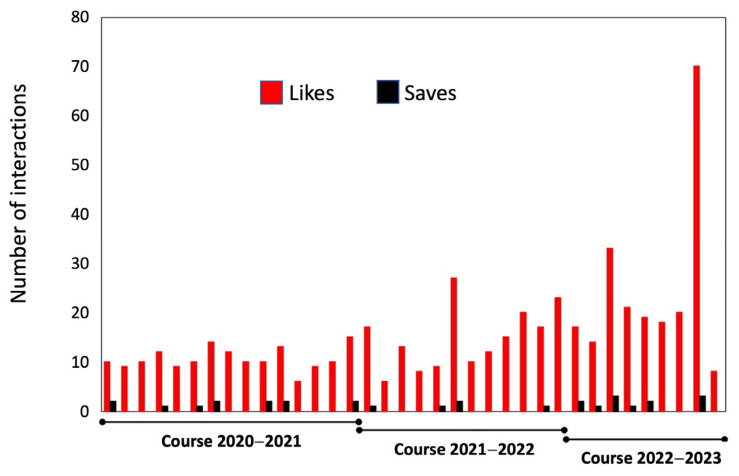
Number of “likes” and “saves” for content shared on the UCOREPRO account over the course of three academic years. Each pair of bars (red and black) in the course timeframe represents one month. It can be observed that the first academic year appears longer because this innovative project and its dissemination activities began earlier. In contrast, the last academic year appears shorter, as data collection concluded before summer holidays.

Figure 2 illustrates the evolution of some Instagram metrics related to the UCOREPRO account. It is evident that between the 2020–2021 and 2022–2023 academic years there is a consistent upward trend in the number of “likes” being awarded to posts. The indicator based on the number of “saves” for each post exhibited a negligible increase.

**Figure 3:**
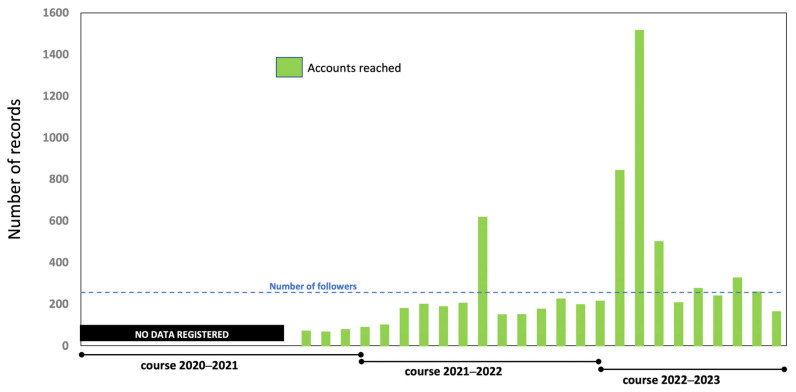
Number of “accounts reached” by the content shared on the UCOREPRO account over the course of three academic years.

Regarding the “accounts reached” metric, which measures the number of unique accounts that have viewed the content on the screen at least once, a significant increasing trend is observed (Figure 3). When using the number of followers at the time of this study as the reference point, it is apparent that there is growing interest in the content over time. This positive trend suggests that the content is successfully attracting attention, and its quality may be improving.

### 3.2. Survey Analysis

The value *ρ_KR_*_20_ = 0.8 derived from the dichotomous questions reveals that the test displays high reliability. The survey was distributed in order to identify the reasons students use social media in the learning environment, particularly the UCOREPRO account, and it was completed by a total of 78 participants. A voluntary response sampling method was employed, aimed at all users of the account. The manager made repeated invitations to students to complete the survey. Nevertheless, the participation rate was relatively low, with only 31% of users (percentage based on the total number of followers at that time) completing it. Most respondents were studying the Veterinary Science course (94.9%), but a small percentage (5.1%) were students on the VET course. None of the master’s students completed the survey.

Approximately 33.3% of respondents said that portable digital devices could replace the use of books, and a similar percentage considered these devices capable of substituting computers in class or at home. Interestingly, 61.5% of respondents reported using portable devices to study and seek information related to veterinary studies. However, a significant portion (69.2%) felt that these devices had limitations, such as the inability to select practice groups during the course or submit assignments, among others.

**Figure 4:**
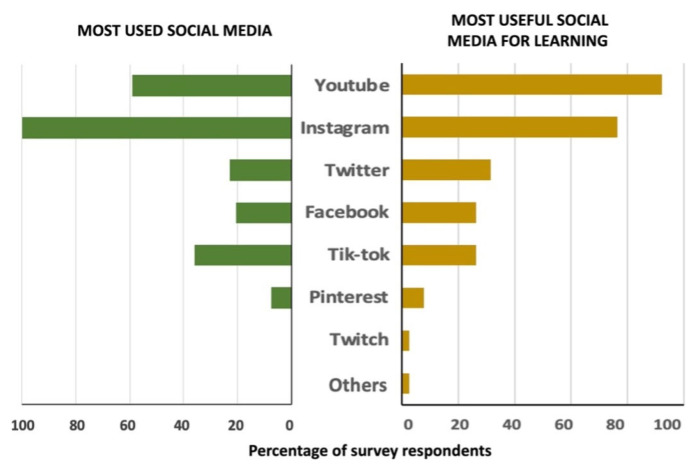
Results of the question, “What social media platforms do you frequently use?” (**left**), and “Which ones do you consider useful for improving the learning process, motivation and academic results?” (**right**).

A high percentage (84.6%) of respondents indicated that these interactive technologies enable them to stay informed about news related to their faculty and university. Approximately half of the respondents stated that they use these media to stay updated on events related to their subject (in this case, animal reproduction) (53.8%) and to seek assistance from fellow students in resolving doubts (51.3%). Others mentioned that social media is valuable for organizing extracurricular activities (41%), reaching out to teachers for inquiries (30.8%), and participating in individual tutorials (10.3%). Only a small percentage (2.6%) of survey respondents reported never using social media for academic purposes. Regarding access to various social media platforms, all participants used Instagram, with many also frequently accessing YouTube. Less frequently mentioned were other social media platforms such as TikTok, Twitter, Facebook and Pinterest (Figure 4). When asked about the most influential and useful social media for improving the learning process, motivation, and academic results, the majority favored YouTube, followed by Instagram (Figure 4).

**Figure 5:**
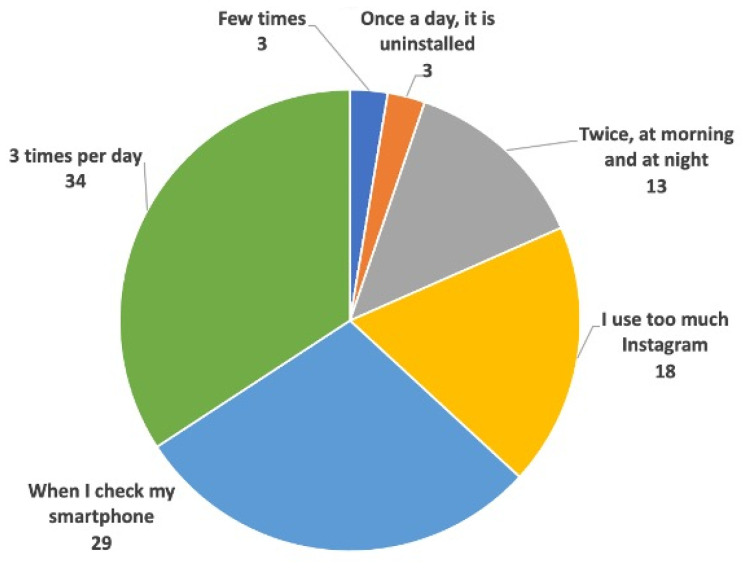
Daily use of Instagram among students (numbers indicate percentage).

The survey also aimed to understand students’ habits related to Instagram usage (Figure 5). Some of them admitted to checking this social media platform excessively (17.9%), while others reported viewing Instagram whenever they use their smartphones (28.2%), three times a day (33.3%), or only in the morning and evening (12.8%). One participant reported uninstalling Instagram from their smartphone in order to save time and reduce distractions. Altogether, 61.5% of survey participants indicated that they spent 3–4 h daily browsing social media, mainly for entertainment content. Additionally, many respondents (71.8%) considered Instagram an effective tool for completing academic tasks or advancing professionally, but they acknowledged that browsing social media also consumed time and incurred distractions.

Regarding the frequency of UCOREPRO posts, students believed that one or two posts per week were desirable, as more frequent publications might lead to fatigue and boredom among users. While 73.7% of survey respondents interacted with UCOREPRO content, a segment of followers and occasional users refrained from active participation due to fears of posting incorrect comments (63.2%) or because they found the content uninteresting (52.6%).

A high percentage of those surveyed (64%) expressed no concerns regarding their privacy.

**Figure 6:**
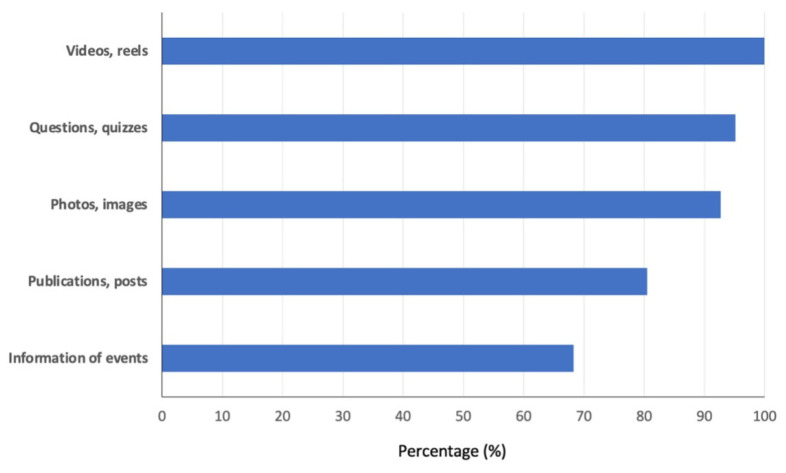
Preferred content types uploaded to the UCOREPRO Instagram account.

The UCOREPRO Instagram account has been active for three years, and users found value in accessing the content created in previous years. Instagram offers diverse content types, with respondents favoring reels (short videos) as their top choice, followed by quizzes or questions about specific themes (in this case, animal reproduction), photos and scientific publications (Figure 6).

While expected benefits for students’ academic success were identified, the survey also highlighted the weaknesses of this initiative. Students noted that because their participation in this activity did not contribute to their final marks in the subject to which the Instagram account was devoted, their interest waned, at least to some extent. Additionally, they indicated that teachers may not be sufficiently well-versed in these technologies, that their privacy could be partly compromised, and that their self-perception was that they were wasting time.

**Figure 7:**
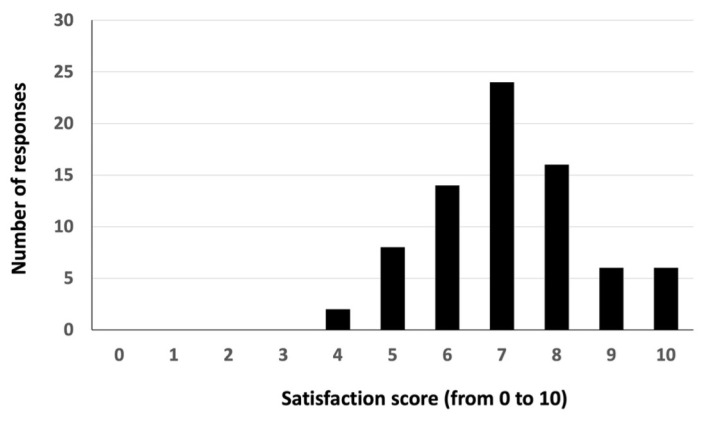
Average satisfaction score among students for the UCOREPRO Instagram account.

UCOREPRO provided students and other users with content related to animal reproduction, with the majority (92.3%) reporting that it caught their interest and attention. Respondents also provided reasons why they viewed this activity positively: the content shared on UCOREPRO facilitated a better understanding of the subject (79.5%), offered new knowledge (71.8%), and motivated students to engage with classroom explanations (53.8%). An overwhelming majority (97.4%) recommended improving the quality and frequency of Instagram posts to enhance audience interest. When asked to rate the activity on a scale of 0 to 10, survey respondents gave it an average score of 7.1 ± 1.5 (mean ± SD) (Figure 7), suggesting that the proposed activity was interesting for students but remains capable of improvement.

It is worth noting that 94.9% of participants believed that this type of social media platform, specifically tailored towards animal reproduction or other veterinary fields, could be beneficial for their future professional networks. And after experiencing the animal reproduction content on Instagram, students expressed agreement with the idea of implementing similar initiatives as teaching and learning tools.

Among the comments provided by the respondents, there were several noteworthy observations that are quoted verbatim in Table 1.

## 4. Discussion

Social media has a huge impact on the life of students, although their use for the teaching–learning process is limited [25]. The present study analyses the implementation of an Instagram account for educational purposes in veterinary studies at the Faculty of Veterinary (University of Cordoba, Spain), where there are no similar initiatives to the best of our knowledge. Data obtained from the Instagram account metrics and the user’s opinions compiled by a questionnaire offered information to achieve a better understanding of the students’ habits on social media and their interest in these digital tools. The implementation of the UCOREPRO account demonstrated an increase in audience attention, mainly due to its ability to enhance understanding of animal reproduction topics and to contribute new knowledge through visual micro-lessons. Additionally, it was considered useful for the development of future professional networks.

The use of portable devices, such as tablets, laptops and smartphones, has become ubiquitous among students. However, the following question arises: are these devices being utilized effectively for learning purposes? In fact, our survey results indicate that 61.5% of respondents use portable devices for studying and accessing valuable information related to veterinary studies, which is consistent with others [9]. A study conducted among students enrolled in medical sciences found that 96.8% use smartphones during classes or meetings, but only 47.3% for more than 10 min for educational purposes, and 95% stated that they use portable devices in the classroom for activities unrelated to medicine [7]. It follows that the development of new educational materials should align with the technological preferences of students in order to improve collaboration among veterinary students [26] and to enhance the overall learning experience [27]. Elsewhere it has been reported that students spend around 1–3 h per day on social media [28]. In the present study, 61.5% of participants said they spend 3–4 h per day browsing social media. Although this could be considered part of students’ leisure time, it may be deemed to be excessive, particularly when some of this time might be redirected towards training and motivational content, relevant to their professional development [29].

The concern about students’ privacy in the context of this academic Instagram account is understandable. The UCOREPRO account is managed by a teacher who would be able to access students’ personal accounts if he followed their accounts. As a result, privacy emerged as a significant concern for students. However, it is noteworthy that 64% of the surveyed students reported no privacy concerns. Social media platforms are designed for virtual social interaction, encompassing various interests, including commercial, entertainment and other purposes. The capacity to access students’ private Instagram accounts raises concerns about the potential intrusion into students’ private lives, as it makes private publications accessible. In the current authors’ view, this could divert from the primary educational objectives of academic Instagram accounts. Because this limitation could impact the growth in followers and the dissemination of educational content, alternative strategies such as the use of effective hashtags and engaging with other educators’ posts should be considered as ways of promoting the learning activity [22].

Determining the ideal frequency for publishing content on an educational Instagram account is challenging. In the case of UCOREPRO, the Instagram account has been active for three years and users have found value in accessing content from previous years, in consonance with others [30]. Short videos have proven to be a positive tool for veterinary students, especially in preparing for practical examinations [31,32]. It has been reported that students receiving video tutorials before classroom sessions exhibit improved performance during practical exercises, underscoring the effectiveness of such educational practices, which is akin to the potential impact of shared content on Instagram [33]. It should be noted that microlearning activities (such as image evaluation, viewing videos, reading texts, etc.) were incorporated into the UCOREPRO account for teaching–learning purposes, and other authors have reported their potential for increasing student motivation, confidence, engagement, knowledge and skills acquisition [34]. However, students also described limitations in interactions with microlearning activities, which may be associated with a fear of being criticized by others; teachers need to be on the alert in order to detect and correct these situations. With the UCOREPRO account, students send their creative content to the account manager (the teacher) for checking, and then improvements and corrections are suggested. Following this, the content is uploaded to the account, either including the student’s identification (or hashtag) or not, depending on their preference. By contrast, other activities in which students are expected to share their personal opinions (as comments) are difficult to promote.

This use of social media as an academic tool comes as a novel approach for teaching staff, but students too are unfamiliar with the practice. One study reports that 87.9% of educator respondents found Instagram increased their sense of self-efficacy, i.e., they consider that they can positively impact student learning, and 80% of them believed it enhanced their pedagogical knowledge [22]. Although social media is widely utilized for educational purposes by medical students [35], its usage among veterinary students remains limited [9], albeit the trend is evolving. Other studies have argued that social networks have a valuable role to play in enhancing educational competencies [36,37,38], which is in line with the present authors’ perception. Previous research into the educational use of social media in veterinary science has concluded that the use of Facebook requires considerable ability on the part of educators if it is to be successful among students [39], whereas using Twitter (now called X) does not ensure that students obtain better academic outcomes [40]. In reference to Instagram, it has been recently highlighted for its potential to enhance veterinary education, specifically when it was used on dairy cow nutrition and management teaching [41]. Be that as it may, students have undoubtedly increased their use of digital devices and social media, which are much more widely accessible, and while the potential advantages remain, this does not lessen the need for appropriate planning on the part of educators. Although rewards in terms of the students’ academic success were anticipated, the survey revealed some shortcomings related to the initiative. The feedback obtained in this study indicates that Instagram users are aware of the potential negative effects of social media on their academic performance, yet they struggle to reduce their usage. Nevertheless, it is evident that students unanimously view social media as a valuable and productive learning tool. Notably, students reported that they would have had more interest in this initiative (the UCOREPRO account) if their participation had counted towards their final subject marks. Furthermore, students pointed out teachers’ lack of familiarity with these technologies, concerns about student privacy and perceived time wastage. Students reported that, while accessing UCOREPRO content on Instagram, they often continued exploring other accounts, particularly those related to entertainment, leading to reduced concentration and potentially affecting their learning experience, which has been described as digital distraction [42,43]. These observations underline a significant gap in awareness and experience in managing social media for educational purposes, at least among educational staff.

Analysis of the survey responses suggests that students appreciated the teaching efforts of their educators to promote activities that can enhance the teaching–learning process. Students commented that the UCOREPRO account increased their motivation towards animal reproduction content, not only among those who created the content but also among those who accessed it. The content created by students and reviewed by teachers represents a new and engaging resource that can be integrated into classroom lessons. By enabling students to develop training materials based on their own practical experiences, this approach encourages more active participation and a shift from a passive to an active role, including taking photos and videos [44,45,46]. It would be unreasonable to expect professional standards of output when students are given digital creative assignments, however. It is also important to bear in mind that the activity of the UCOREPRO account is designed to motivate students to learn the content they are already working on. Five approaches for using virtual learning environments have been suggested [47], and the implementation of this Instagram account for educational purposes can be aligned with category E, which is defined as “a collaborative-focused strategy with the intention of providing an online space for building knowledge”. In general, social media posts incorporating images and videos have a greater chance of being used by students, thereby helping to develop their abilities and knowledge base [48].

Current e-learning platforms are more rigid than social media. Moodle, for example, is a learning platform used by students over the course of their academic studies, but once these have come to an end, they will never access it again. Social media by contrast is intimately woven into students’ lives, enabling an unrestricted style of on-demand learning, and making content available for an indefinite length of time. Internal college learning systems offer certain advantages, including robust privacy controls and protection from the data security risks commonly associated with public media platforms. For its part, the UCOREPRO account provides unique teaching benefits like easy mobile access, engaging visual content, and interaction among peers that significantly increased student interest and motivation. Specifically, by delivering microlearning activities, Instagram enhanced the teaching–learning process by catering to varying attention spans and promoting informal, on-demand learning beyond scheduled class times. Moreover, its community-building features (stories, reels, direct messaging) enable collaboration and knowledge exchange across veterinary schools and geographically isolated areas, a reach that an isolated internal platform typically cannot match. To combine the best of both worlds, an internal system could be modified to incorporate Instagram-style benefits—embedding a mobile-friendly social feed, supporting short video modules, facilitating peer content creation, and delivering real-time notifications—while maintaining institutional privacy safeguards and centralized data management. In a world where education is becoming increasingly digital and interactive, the importance of continuing education and keeping abreast of new digital technologies is clear [49]. It is important for educators to occupy a space in this digital environment. If the virtue of technology is to create learning experiences in a range of contexts to engage the student, such contexts should be harnessed to demonstrate the use of meaningful and innovative proposals that succeed in spanning formal academic learning and its informal counterpart.

The UCOREPRO account is currently operating, and it will be available in subsequent academic years, during which the acquired experience will enhance it. In spite of the relatively low number of people following the UCOREPRO account, there needs to be a discussion about what the target audience should be; while this account is theoretically open to view by any user of the Instagram app, if specific technical content is uploaded relating to a particular animal, it would be advisable to ensure it is not visible to the owner of the animal concerned or any other non-professional viewer.

Instagram represents a promising tool for reaching underserved or geographically isolated communities, particularly in the field of veterinary education. Its accessibility, visual nature, and widespread use among younger generations make it a valuable platform for sharing educational content beyond traditional institutional boundaries [50,51]. By leveraging Instagram, veterinary educators can disseminate knowledge, showcase clinical cases, and promote best practices in a format that is both engaging and easily accessible. Furthermore, the platform can foster new opportunities for collaboration between veterinary schools across regions and countries, enabling the exchange of ideas, joint educational projects, and increased visibility for initiatives that might otherwise remain localized.

In light of the responses from the participants and based on our experience, several measures have been identified that could enhance this project: (a) Offer educational incentives to students who produce high-quality educational content, such as an increase in their final academic marks. (b) Establish a system for reciprocal and automatic tracking of all students who follow the account, increasing the reach of educational content and its potential benefits. (c) Enhance the digital skills of the teaching staff, particularly in relation to social media management. (d) Develop a consistent schedule for publishing educational content on the Instagram account, such as twice a week, to establish a more predictable routine for followers. (e) Create new strategies for collecting feedback from followers, as surveys may not be engaging for them. (f) Use social media to disseminate scientific material in an openly available and informal way to students who are less familiar with scientific discourse.

Limitations related to the small sample of participants who completed the questionnaires and the bias associated with the voluntary nature of the surveys were identified in this study. Although participation was optional to avoid contributing to students’ survey fatigue—an increasingly common issue in academic settings—the low response rate highlights the need to develop new dissemination strategies to reach a broader audience and promote engagement. In the current university context, students are frequently required to complete numerous surveys for each course, teacher, or subject, which may result in survey fatigue and reduced motivation to participate in additional studies [52]. Graduates and clinicians may also encounter similar survey demands, adding to their academic and professional workload. Despite the limited sample, the feedback received was considered valuable, as it likely came from participants who were genuinely interested and willing to engage critically. Their input provided meaningful insights to evaluate the initiative, understand user needs, and improve the educational tool. However, it is important to acknowledge that non-mandatory web surveys may yield biased estimates by excluding students who are less active or not interested in using these technologies for learning purposes [53].

## 5. Conclusions

Nowadays, social media platforms are frequently accessed by learners for leisure purposes. A lot of time is spent browsing their content. Instagram is one of the most popular social media sites, and it offers an effective platform for developing microlearning activities capable of enhancing the teaching–learning process. However, students do not necessarily associate such platforms with educational purposes, nor are teachers familiar with them as didactic tools. The survey shows that smartphones and other portable devices are frequently used for study and to undertake learning activities, and it also reflects the high interest of students in social media as an educational tool. The UCOREPRO account earned an average rating of 7.1 out of 10 in terms of satisfaction. In order to take advantage of the value of Instagram for education, some issues need to be overcome, including privacy, low participation, absence of academic rewards and restricting access exclusively to target groups. Students reported numerous positive features of UCOREPRO, including its capacity for engendering their interest and attention in animal reproduction, facilitating understanding of the curriculum, providing new knowledge, motivating them to engage with the teacher’s explanations, and connecting with professional networks. Another point to be borne in mind is that educational Instagram accounts provide information to students around the world, with different personal profiles but similar levels of inquisitiveness.

## Figures and Tables

**Table 1:** Users’ personal opinions of the UCOREPRO account drawn from the survey.

Student	Opinions
1	“I think it would be very interesting to announce in advance when the contents will be uploaded, since they often get lost on Instagram.”
2	“Higher dissemination and impact of this account could attract more student attention.”
3	“I really like this initiative because it encourages using social media for learning. Therefore, I believe more content should be posted, as it motivates followers to participate and become interested in these topics.”
4	“I really liked knowing that some professors make an effort to connect with students, trying to use new tools for them. But social media (like Instagram) has many disadvantages. And there are already many of us spending too much time on our phones (several hours a day). I’m not sure if further promoting the use of these platforms (which can bring many problems to young people), even for an academic activity, is a good idea. Furthermore, some people don’t want to use social media too much. But in general, the idea seems very healthy for Instagram users, and those who like to use social media to learn things…”.
5	“I consider it a very good initiative with great untapped potential. As a student, I believe that many times we don’t interact with this type of account because we are afraid of saying some crazy or wrong idea.”
6	“It would be useful to post more frequently and on different topics, in order to help students with the subject.”
7	“In my opinion, it fosters a lot of interest in the subject and contributes to a better understanding of the classes and the contents. It could offer educational advantages for students, and it is a new, more fun, entertaining and participatory way to acquire knowledge about the subject.”
8	“I think teachers should invest in this kind of activity because, amid all the useless content, we can find interesting and useful information while we are taking breaks, which captures our attention.”
9	“It’s a very visual, schematic and attractive tool for students. If real clinical cases or very simple explanations are shared this way, it can make a big difference to reinforcing concepts and training material…”
10	“I think it’s great that teachers are so involved and make an effort for us to learn in such an engaging and dynamic way. And using Instagram for this purpose is innovative and original. I hope there are more subjects like this.”
11	“It’s a good initiative, but we tend to forget it exists, or we are not so used to using social media (in this case, Instagram) for academic purposes. But it’s very interesting, and in my opinion, we should participate more in UCOREPRO.”

## Data Availability

The original survey used in this study is included in the Supplementary Materials. The data obtained are available on request from the corresponding author.

## Supplementary Materials

The following supporting information can be downloaded at: https://www.mdpi.com/article/10.3390/vetsci12080698/s1, Table S1: Survey questions.
