# Supplementary material for: Use of Instagram as an Educational Strategy for Learning Animal Reproduction
Source: Vet Sci. 2025 Jul 25;12(8):698. doi: 10.3390/vetsci12080698 (PMC12390405; doi:10.3390/vetsci12080698)
Supplement: Supplementary file 1 [file vetsci-12-00698-s001.zip › vetsci-3701248-supplementary.pdf]

veterinary sciences

MDPI

Suplementary Table S1. Survey questions.

SURVEY

1. Are you students of the Veterinary Science degree, Master's degree, other? (Y/N)

SMARTPHONE

2. Can portable devices (smartphones, tablets, laptops) replace conventional books? (Y/N)

3. Can portable devices replace computers? (Y/N)

4. Can you study by smartphone or tablet? (Y/N)

5. Do you think that smartphones are a good tool for searching for information related to Veterinary Science? (Y/N)

6. Can you do all the activities required in class (select practice groups, send homework, study...) using only a smartphone? (Y/N)

SOCIAL MEDIA

7. What social media platforms do you frequently use? Facebook, Instagram, Pinterest, TikTok, Twitter, YouTube, other.

8. Which ones do you consider useful for improving the learning process, motivation and academic results? Facebook, Instagram, Pinterest, TikTok, Twitter, YouTube, other.

9. How much time do you spend using social media per day?
One hour
Around 3-4 hours
More than 4 hours
I think I use social media too much
Other

10. Do you think social media are a good alternative for learning about animal reproduction? (Y/N)

11. Can you do homework using information obtained from social media? (Y/N)

12. Do you think that social media give you too much unreliable information? (Y/N)

13. Do social media disrupt students despite helping them in the learning process? (Y/N)

14. Do you like to use social media for academic tasks? (Y/N)

15. Are you worried about your privacy on social media? (Y/N)

16. What do you use social media in the academic environment for?

I never use them for academic purposes

With other students, to resolve queries/uncertainties

To do homework

To ask teachers questions

To keep updated about the subject

To read news about my university or faculty

To organise extra-curricular activities

Other

INSTAGRAM

17. Do you have an Instagram account? (Y/N)

18. How often do you check your Instagram account?

Three times per day

When I check my smartphone

Twice, in the morning and at night

I think I use Instagram too much.

Other

19. Is Instagram interesting as an information resource for animal reproduction content? (Y/N)

20. Is Instagram useful for promoting student-teacher contact? (Y/N)

21. Can Instagram be useful for obtaining clients in your future professional activity? (Y/N)

22. Are you a regular follower of the UCOREPRO account or do you only access to the account sporadically? (follower/sporadic)

23. Do you interact with the content on the UCOREPRO account and why? (Y/N) Give your opinion.

24. Has UCOREPRO increased your attention and/or interest in the subject of animal reproduction? (Y/N)

25. What can be done to promote this account among students?

Nothing

Add more and better activities

Offer new knowledge

Offer better understanding of content

Other

26. Do you think that this account (devoted specifically to animal reproduction content) can help you to obtain professional contacts or relationships for your future career? (Y/N)

27. Do you think this kind of tool, i.e. social media, should be used for the learning-teaching process? (Y/N)

28. Are more activities or content required for this account? (Y/N)

29. How often should content be uploaded to the account to secure students' attention?

Daily
Once per week
Twice per week
Once per month
Other

30. Why do students not interact with the UCOREPRO account?

They do not find it interesting
They would not like other students to think that they are overly participative
They are afraid to give incorrect answers.
Other

31. Have you checked posts from previous academic years on the UCOREPRO account? (Y/N)

32. Is it valuable to preserve posts from previous academic years? (Y/N)

33. What type of information should be shared on the UCOREPRO account to increase students' interest?

Photos/Images
Videos
Scientific publications
Questions, quizzes
Publicity about specific events
Others

34. What disadvantages are there of using Instagram in the learning-teaching process?

It is not considered for the final marks
Teachers are not technologically up-to-date
Students lose privacy
Students waste time
Other

35. Please rate from 0 to 10 your experience of using the UCOREPRO account.

Finally, please add any information or opinions that could be useful for enhancing this tool; it is very important for us!
